# Measuring the Impact of Incorporating Case Study Presentations Into Applied Biomedical Science Placement Workshops for Trainee Biomedical Scientists

**DOI:** 10.3389/bjbs.2024.12017

**Published:** 2024-02-20

**Authors:** Amreen Bashir, Kathryn Dudley, Karan Singh Rana, Kayleigh Wilkins, Ross Pallett

**Affiliations:** ^1^ School of Biosciences, College of Health and Life Sciences, Aston University, Birmingham, United Kingdom; ^2^ Faculty of Science and Engineering, Wolverhampton School of Sciences, Wolverhampton University, Wolverhampton, United Kingdom

**Keywords:** IBMS registration portfolio, biomedical science, HCPC SOPs, case study presentation, collaborative working

## Abstract

**Introduction:** Successfully completing the Institute of Biomedical Science (IBMS) registration portfolio is essential to becoming a Health and Care Professions Council (HCPC) registered Biomedical Scientist. In the West Midlands, a unique collaboration between four universities (Aston, Wolverhampton, Coventry, and Keele) and local NHS Trusts supports student placements and portfolio development. The universities support Training Officers in delivering components of the registration portfolio through the delivery of eight combined placement workshops. These have been designed to align to the IBMS registration portfolio and help students meet the HCPC Standards of Proficiency. This study aimed to evaluate the effectiveness of a redesigned workshop where students generated and presented medical case studies to peers, academics, and training leads.

**Materials and Methods:** The three phases of the case study intervention included a pre-intervention survey, academic-led sessions focussing on medical case presentations and delivery of the presentation followed by a post-intervention survey.

**Results:** Analysing survey responses pre- and post-intervention, students demonstrated enhanced confidence in their understanding of clinical conditions (*p*<0.0001), connecting lab findings to diseases, and in delivering a case presentation to their peers (*p*<0.001). Students reported an increased confidence in structuring case presentations and their critical thinking ability (*p*<0.0001). All students agreed engaging with the case study workshop improved their ability to communicate knowledge of scientific concepts orally. Thematic analysis revealed that the case presentation deepened students' understanding of multidisciplinary teams. 98% of respondents agreed patient communication should be integrated into Biomedical Sciences courses and 85% would like to see case study presentations embedded into the curriculum.

**Discussion:** Combined placement workshops are an integral part of the Applied Biomedical Science placement journey. Case study presentations are clearly a valuable teaching and learning tool to nurture and develop key transferable skills and competencies in conjunction with Biomedical Science expertise. The collaborative approach in the West Midlands effectively prepares graduates with essential pathology knowledge, skills, and a completed IBMS registration portfolio. This study highlights a successful framework for a collaborative partnership with local NHS trusts that has allowed the completion of numerous pathology placements and could be adopted by other universities delivering accredited Biomedical Science courses.

## Introduction

To work as a Biomedical Scientist within an NHS laboratory, individuals must be registered with the Health and Care Professions Council (HCPC) [1]. Candidates must complete an Institute of Biomedical Science (IBMS) registration training portfolio in an IBMS approved training laboratory. This allows the candidates to demonstrate that they meet the HCPC Standards of Proficiency (SOPs) [2] and have developed the knowledge and skills required to work as a state registered Biomedical Scientist. Following a successful portfolio verification, individuals can be awarded the IBMS certificate of competence, enabling candidates to register with the HCPC.

There are two main routes for UK candidates wishing to complete their IBMS registration portfolio. The first route entails completing a 3-year IBMS accredited Biomedical Science undergraduate degree programme and seeking a trainee Biomedical Scientist position post-graduation. The second route involves the candidate completing an integrated placement year at the end of the second year of study. The Applied Biomedical Science route allows students to complete their IBMS portfolio prior to graduating [3].

Globally, it is well known that obtaining a placement within a medical setting can be difficult [4]. Reasons behind this difficulty can be due to medical workforce shortages, a lack of training leads, and high workloads impacting training delivery and capacity [5]. Specifically, within the UK, the NHS is facing its largest staff shortage in recent years with a need for fully qualified staff [6]. The pressure to hire fully qualified staff limits the number of available trainee Biomedical Scientist positions available to graduates. This makes the Applied Biomedical Scientist placements very competitive and are regarded as being highly prestigious.

### The Role of a Successful University-Pathology Partnership for Placement Delivery

The relationship between higher education institutions (HEIs) and local NHS laboratories varies across the United Kingdom. A recent survey was distributed via the practice educator network to NHS pathology laboratories which sought to collect data related to graduate employability skills [7]. A major finding of this study identified that only 52% of pathology laboratories were involved in university employer liaison groups. Through the data, it was highlighted that some liaison groups were only meeting annually to discuss placement opportunities. Furthermore, comments were made about graduates not having a sound understanding of the importance of laboratory accreditation through the United Kingdom Accreditation Service (UKAS) and the essential role of pathology services in providing patient care [7].

As a Higher Education provider, the key to obtaining trainee Biomedical Scientist placement posts for students is creating a close relationship with local laboratory managers and practitioners [8]. Within the West Midlands, there is a unique partnership between four HEIs (Aston University, Wolverhampton University, Coventry University and Keele University) and the local NHS Trusts. This partnership was initially forged by Yvette Taylor, a Senior Biomedical Scientist and the training lead at Birmingham Children’s and Women’s Hospital. Yvette was central to creating links between local hospitals and universities, to establish the West Midlands Training Officer (WMTO) group, a bi-monthly forum to facilitate training-related discussions. Furthermore, these universities and hospitals are involved in a University Employer Liaison Committee (UELC) which meets at least three times a year to discuss placement student performance, portfolio progress, verification and future placement opportunities, in addition to tailored support provided by the universities in response to Training Officer needs. In order to further support Training Officers, each of the universities involved in the UELC host a Train the Trainer (TTT) day. Programmes have included portfolio verification training for new verifiers, discussion of appropriate evidence pieces and changes to HCPC SOPs [2]. An element of this partnership has been to support Training Officers in delivering components of the registration portfolio thus making this a “symbiotic relationship,” as all parties are supporting each other equally [8]. The four universities deliver a series of combined placement workshops that have been designed to align to the IBMS registration portfolio and help students meet the HCPC Standards of Proficiency.

### University Support Provided for Placement Students

The trainee Biomedical Scientist placements offered by the NHS Trusts within this unique partnership are specifically open to students from five universities, following the recent addition of Staffordshire University. The process has been streamlined over several years, with Aston, Wolverhampton and Coventry universities having contributed to the process for student applications (including the design of the application form), pre-placement laboratory audit form and combined placement workshop programme. Currently, the combined placement workshops are compulsory for students enrolled across all universities within the partnership. Students benefit from a collaborative delivery approach, whereby the universities share the delivery of the workshops to support the ∼30 students whilst on placement.

Students wishing to undertake trainee Biomedical Scientist placements are mentored through a series of pre-placement workshops to build the skill set required to work in pathology laboratories [7]. These include CV writing, placement application support, and mock interviews. Students are further supported with core skill development through attending pre-placement laboratory workshops, including microscopy, pipetting, preparing solutions, carrying out dilutions and basic diagnostic testing [9]. Students are also provided with a session led by local Training Officers which covers professional conduct and requirements of a registrant, dealing with sensitive data, patient confidentiality and effective communication. These pre-placement workshops are delivered by individual HEIs in November with students applying for placements with local NHS trusts the following March/April to begin the sandwich placement in September.

### The Significance of Developing Case Studies Within Biomedical Science

**FIGURE 1:**
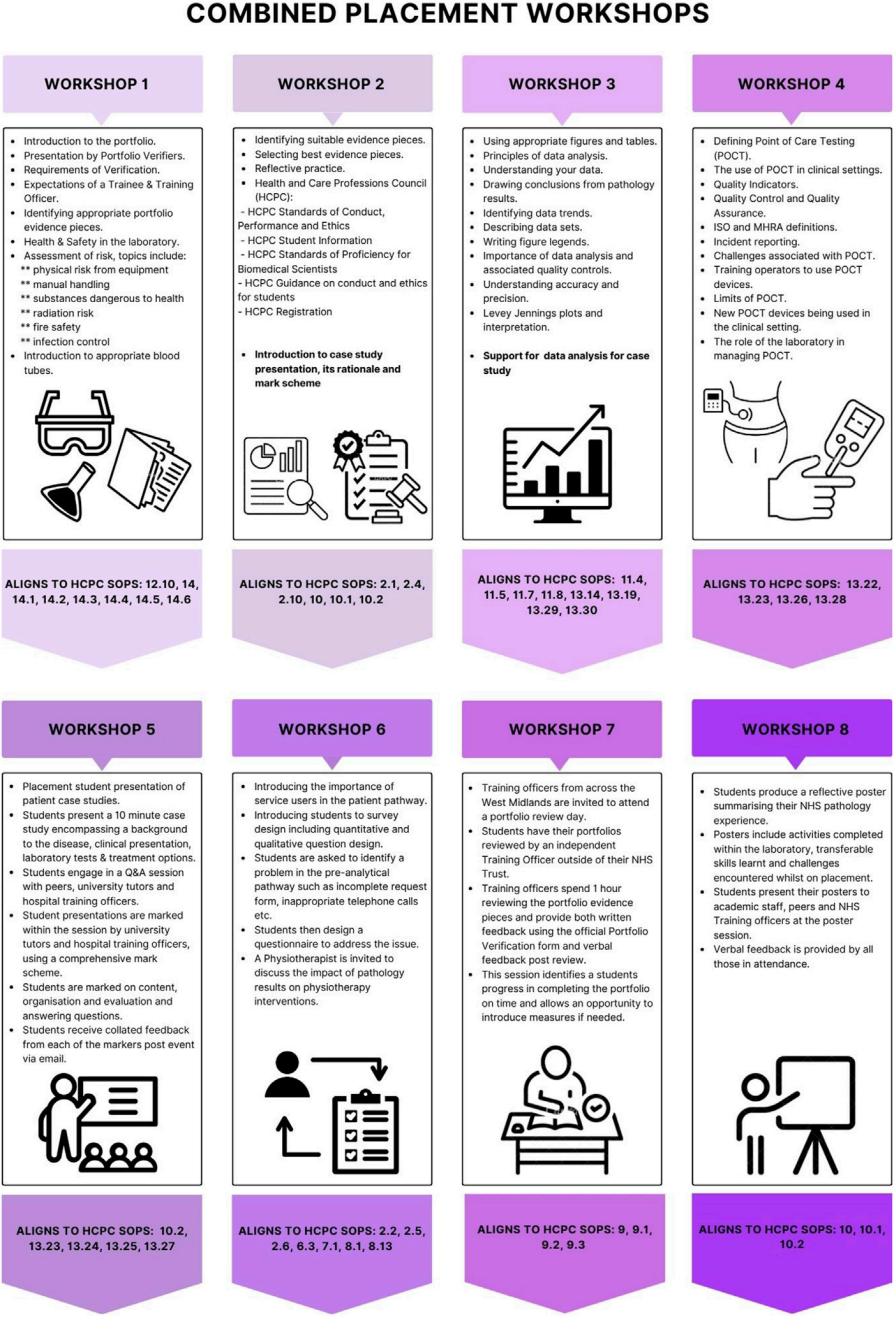
Details of the eight combined placement workshops delivered across the placement year to support the completion of the IBMS registration portfolio with corresponding 2023 revised HCPC SOPs for Biomedical Scientists.

Originally, the HEIs delivered six combined placement workshops. Recently, a further two workshops have been introduced to include case study preparation to further align to the IBMS registration portfolio and help students meet the revised 2023 HCPC SOPs [2, 3] (Figure 1).

The first two workshops include a range of topics such as introducing students to the registration portfolio, expectations of HCPC registered professionals, health and safety in the laboratory, and appropriate evidence pieces. Workshop 3 focuses on data analysis and interpretation, whilst workshop 4 centres around point of care testing (POCT) and quality assurance. Workshop 5 requires students to present a patient case study to their peers, training officers and academics. Workshop 6 focuses on the central role of service users, focusing upon a specific example of how a physiotherapist utilises diagnostic laboratory test results to treat patients. Workshop 7 provides an important opportunity for training officers to review portfolio progress. As part of this workshop, students are required to bring their portfolios and an external training officer provides feedback on evidence pieces. The final workshop in the series requires students to present a poster which summarises their placement experience (Figure 1).

### Further Embedding Case Studies Into the Biomedical Science Curriculum

One key factor that has been emphasised throughout the redesign is to further embed the significance of understanding case studies within Biomedical Science. Prior to re-designing the case study workshop, students were required to independently construct and present a case study presentation at one of the host universities. Historically, feedback from the Training Officers who marked the case study presentations demonstrated varying levels of success and engagement from students. For example, some presentations failed to discuss the laboratory tests, how they are performed and the importance of the results on the patient pathway. Another issue highlighted pre-design of the workshops was students expressed transport difficulties to attend one of the host universities within the West Midlands region. This meant that some students could not attend the entire workshop and ultimately missed out on this educational experience. To address these issues and better support all students, the case study combined placement workshop was redesigned to be delivered virtually and more structure was provided for constructing and presenting medical case studies.

The aim of this study was to evaluate the success of preparing trainee Biomedical Scientist placement students to effectively construct and evaluate a medical case study, that was delivered to a virtual audience of student peers, academics and training leads. The survey sought to determine whether the format was effective in deepening a student’s understanding of the importance of pathology services within the delivery of a multi-disciplinary team working together for modern patient care.

## Materials and Methods

### Placement Opportunities Within the West Midlands

Each year the number of placement opportunities for trainee Biomedical Scientists in the West Midlands varies depending on the laboratory training capacity. Table 1 shows the number of positions that have been available over the past 3 years. The four universities put on a series of combined placement workshops throughout the year to support Training Officers and trainee Biomedical Scientists to complete the IBMS registration portfolio. These workshops are delivered to all students across the four universities and have compulsory attendance.

**TABLE 1:** Placement opportunities within the West Midlands between 2020 and 2023, and survey completion rates.

Year	Number of trainee BMS positions in the West Midlands	Total number students who completed the survey
2020–21	20	12 (70%)
2021–22	19	17 (90%)
2022–23	30	19 (63%)

### Case Study Workshop Re-Design

To improve understanding of disease conditions, laboratory diagnosis and treatment this pedagogical intervention was introduced in the year 2020. Students who choose to participate were required to read a Participant Information Sheet and complete the online consent form prior to taking part in the study. Ethical approval for this survey was covered by the original three universities in the West Midlands working group (Aston University Ethics #1734, Wolverhampton University Ethics #3693 and Coventry University Ethics #1258). Throughout the 3 years, responses from participants across the now five contributing universities have been gathered.

**FIGURE 2:**
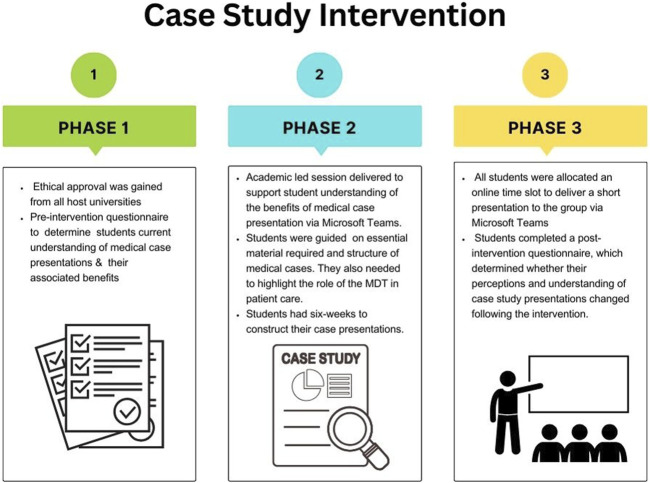
The three phases of the case study intervention. This includes a pre-intervention survey, academic-led sessions focussing on medical case presentations and delivery of the presentation followed by a post-intervention survey.

The research was conducted in three phases. In Phase 1, we determined the students’ current understanding of medical case presentations and their associated benefits through the completion of a pre-intervention questionnaire using the Online Survey tool [10] (Supplementary Material S1). In Phase 2, a session was delivered to support students’ understanding of the benefits of medical case presentations and to support the development of their own medical case presentations via Microsoft Teams. Students were required to include disease aetiology, laboratory tests performed to support diagnosis and the specific role of the student in the diagnosis pathway. Students needed to discuss the laboratory results for the patient, in addition to treatment. Students also needed to highlight where relevant the role of the multidisciplinary team in patient care. Students then had a 6-week period to construct their case presentations, where they were able to gain support from university academics and their training leads. In Phase 3, all students were allocated an online time slot to deliver a short presentation to the group via Microsoft Teams (5 min presentation and 5 min for questions and comments from the audience). Finally, students were invited to complete a post-intervention questionnaire, which determined whether their perceptions and understanding of case study presentations changed following the intervention (Figure 2).

### Training Officer Feedback

Pathology Training Officers who recruit students through the West Midlands Applied Biomedical Science Placement process were invited to provide feedback on the combined placements workshops and training provided by the universities.

### Data Analysis

Quantitative data gathered from the case study evaluation survey was reported as mean ± standard error of the mean and Median. Likert Scale responses were converted to a numeric format (4 = confident, 3 = slightly confident, 2 = somewhat confident and 1 = not very confident) prior to statistical analyses using non-parametric methods. For all pre and post case study data, a Wilcoxon signed-rank test was conducted using IBM-SPSS Statistics version 26. Statistical significance was set at *p* < 0.05. Free text comments were analysed via thematic analysis using the Braun and Clark Framework to identify significant themes [11].

## Results

A total of 48 students who engaged in the case study workshop completed the pre and post survey over 3 years [2020–21 (*n = 12*), 2021–22 (*n = 17*) and 2022–23 (*n = 19*)]. All survey responses were anonymous to avoid any form of bias (Table 1).

### Free Text Responses for Thematic Analysis

#### Pre-Survey

In the pre-case study workshop, students were asked what skills they thought were necessary to effectively deliver a case study presentation. Student responses included confidence, public speaking, oral and written communication, maintaining eye contact, time management, organisation, active listening, and critical thinking.

**FIGURE 3:**
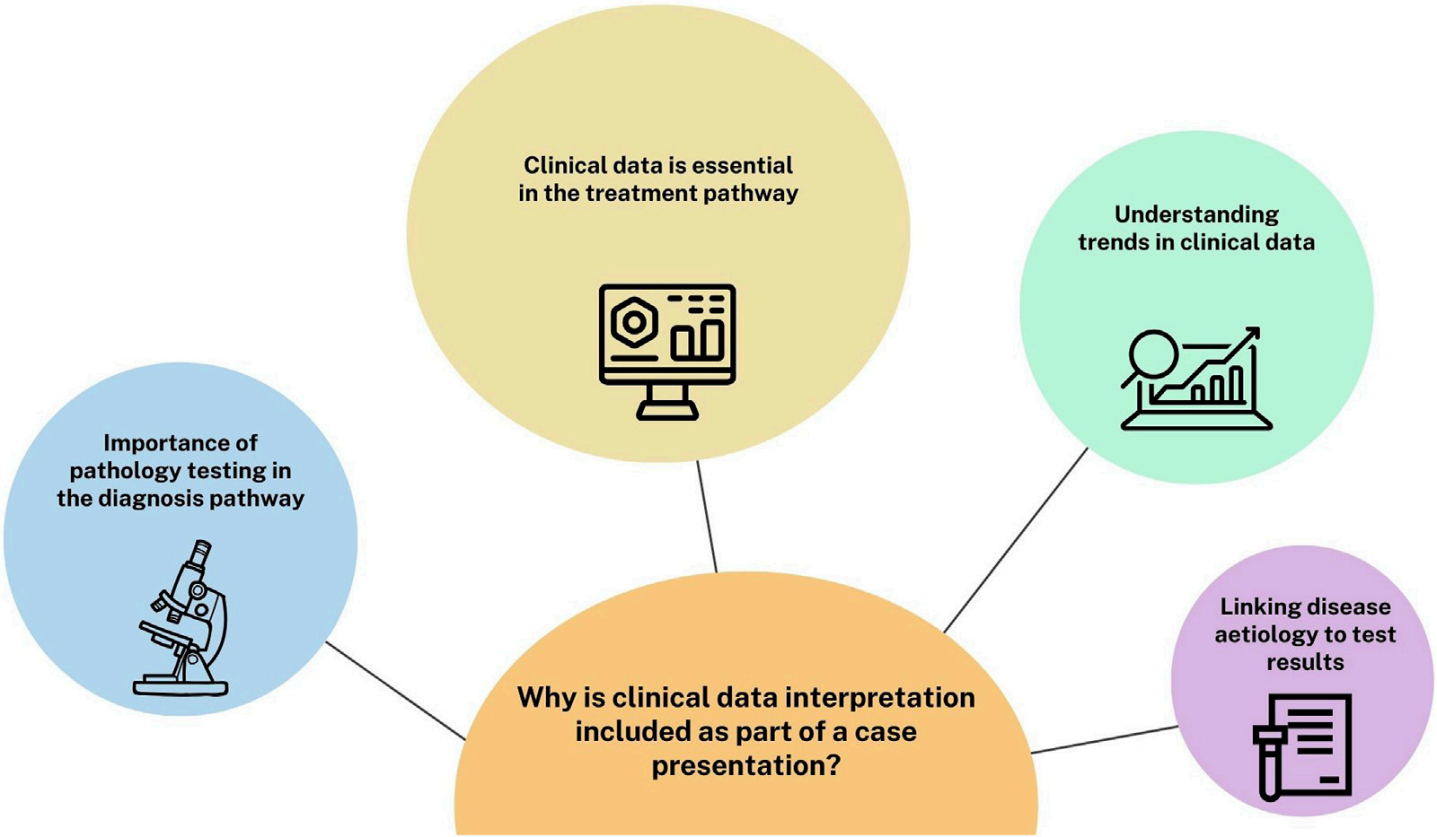
A thematic analysis of open-text responses to the importance of clinical data interpretation as part of case study presentations. This question was included in the pre-intervention survey. Four major themes were identified, and the occurrence of each theme is represented by the size of each circle in the schematic. Several students open-text responses contained more than one theme.

A free text option question was included to gauge a better understanding of the importance of the need for clinical data interpretation as part of case study presentations, (Q23). In total, 77% (*n = 44*) of the students answered question 23. Some student responses fit into multiple themes and once the responses were analysed the four finalised themes identified were categorised as shown in Figure 3. The most prominent themes identified were:

1. Clinical data is essential in the treatment pathway.
2. The importance of pathology testing in the diagnosis pathway.
3. Understanding trends in clinical data.
4. Linking disease aetiology to test results.

Specific comments included:

“As a Biomedical Scientist it's important to have a good understanding of how the data relates to a patient and the care they receive”

“Clinical data interpretation is included to show how medical professionals reach the conclusion of a specific condition/disease/syndrome. It also provides knowledge to those listening to the presentation who may not be aware a syndrome/disease/condition”

“It is important to analyse the findings in patient results and linking this to the clinical details of the patient. To interpret clinical results, the case study can come to a possible conclusion with the type of disease being identified and methods of treatment prescribed to the patient to relieve their symptoms”

“Clinical data interpretation is included as it gives greater context to the case, being able to visualise severity and relate this to the aetiology and the patient”

“Interpretation enables the audience to gather an idea of how results link to diagnosis and disease pathophysiology. Applies knowledge to clinical situation”

**FIGURE 4:**
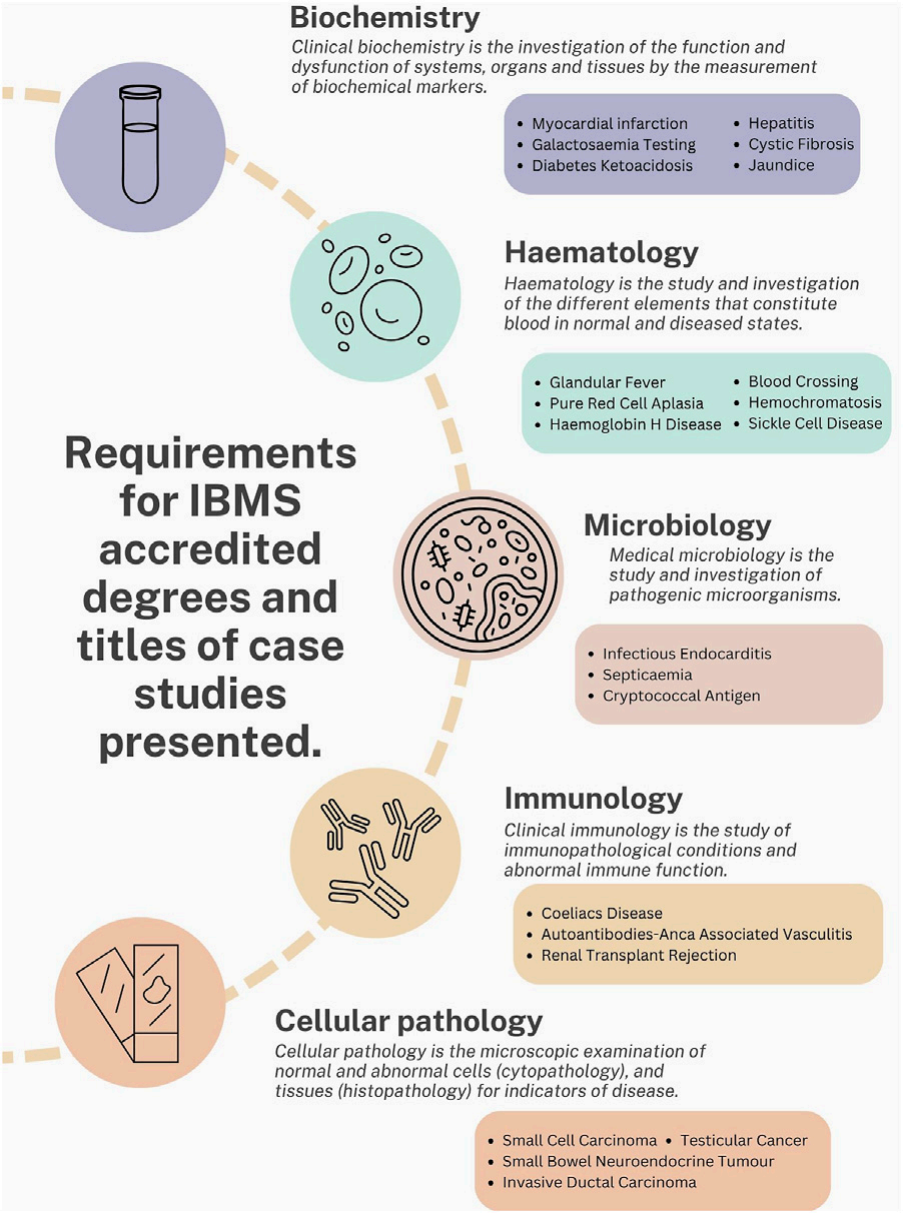
Examples of clinical conditions covered by students during case study presentations. These are presented with the relevant disciplines the students completed their placements in, which are also mapped against the IBMS requirements for graduate learning on an IBMS-accredited program.

The case study presentations included a wide range of clinical conditions, linked to the discipline the student undertook their placements in. Many of these conditions students would not have encountered in such depth during their undergraduate studies prior to placement. Examples of such conditions include Small Bowel Neuroendocrine Tumour; Autoantibodies-ANCA Associated Vasculitis and Pure Red Cell Aplasia. These clinical case studies are relevant to pathology disciplines that students must study to graduate from an IBMS-accredited degree. Students have a limited opportunity to rotate to other disciplines during their placement year and this case study workshop created an opportunity to learn about multiple pathology disciplines and health conditions in one session (Figure 4). Assessors were from a range of backgrounds with representatives from Biochemistry, Haematology, Immunology, Cellular pathology, and Microbiology.

#### Students Report an Increase in Transferable Skills Post-Workshop

Students were required to complete two short surveys, one prior to the case study workshop and one following their case study presentation. Student responses were coded into scores (4 = confident, 3 = slightly confident, 2 = somewhat confident and 1 = not very confident) and reported relative to their perceived confidence prior to engaging with the case study workshop.

Students self-reported an increase in confidence in their understanding of a range of diseases/disorders diagnosed in the laboratory and linking test results produced in the laboratory to diseases/disorders (*p* < 0.0001). Furthermore, students reported feeling more confident in giving a case presentation to their peers, (*p* < 0.001) as well as their overall confidence in public speaking (*p* < 0.05). Students demonstrated increased confidence in their ability to structure a case presentation and their confidence to think critically (*p* < 0.0001).

**FIGURE 5:**
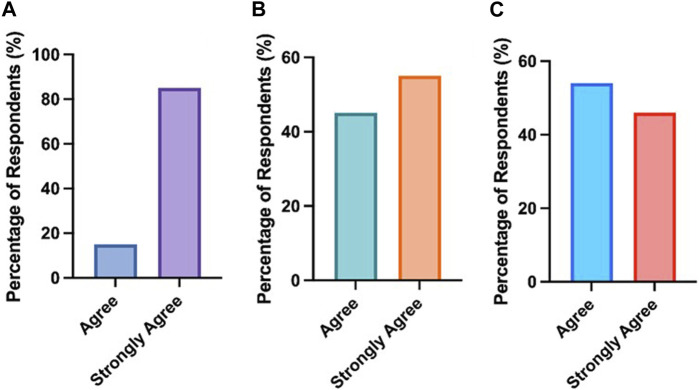
All students agreed that engaging in the case study workshops enhanced **(A)** their ability to communicate knowledge of scientific concepts orally, **(B)** their understanding of the underlying biological basis of the disease and **(C)** helped them establish the link between theoretical content and practice.

As part of the post-case study presentation survey, students were asked a series of questions. Most notably, if they felt engaging with the case study workshop improved their ability to communicate knowledge of scientific concepts orally. A total of 85% of respondents strongly agreed and 15% agreed with this statement. Furthermore, all students (100%) agreed or strongly agreed that working through the case study workshop improved their understanding of biological basis of disease. Finally, 46% of respondents strongly agreed and 54% of respondents agreed that following the completion of the case study workshops, students felt better placed to establish links between theoretical content and clinical practice. As shown in Figures 5A–C, all respondents (100%) either agreed or strongly agreed that the workshop improved these areas.

**FIGURE 6:**
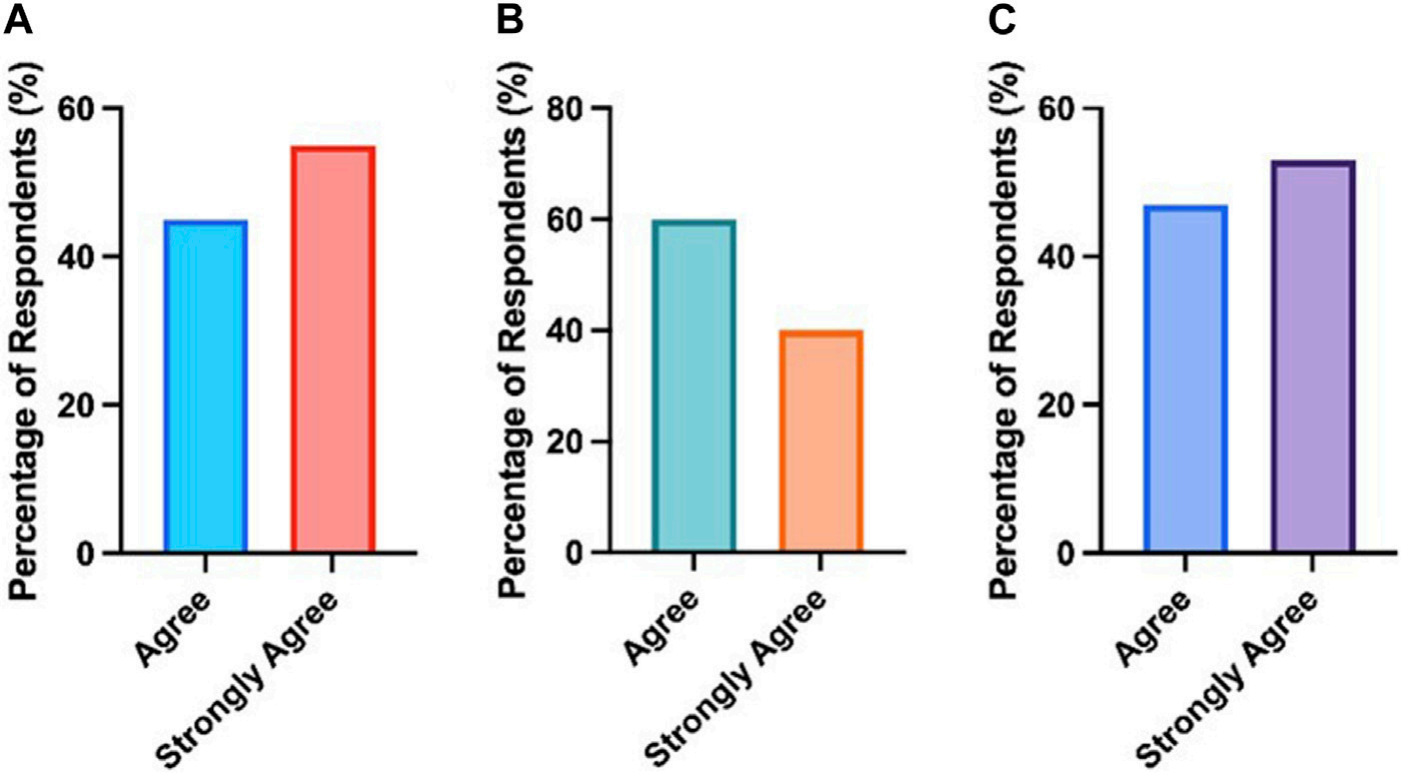
All students agreed that engaging with the case study workshops enhanced **(A)** their understanding of the topic using online resources/books/review papers **(B)** their development of reflective and critical thinking and **(C)** understanding of the whole patient pathway from sample collection and processing to the treatment of the patient.

The case study workshop was successful in developing skills in using scientific resources, reflective thinking and clinical patient pathways**.** Additionally, 55% of respondents strongly agreed and 45% of respondents agreed that the case study presentation opportunity deepened their understanding of the topic through scientific sources. All students (100%) agreed or strongly agreed that the case presentation enhanced their reflective and critical thinking. Finally, all students (100%) agreed or strongly agreed that following the completion of the case presentation they had a better understanding of the whole patient pathway from sample collection and processing to the treatment of the patient. As shown in Figures 6A–C, all respondents (100%) agreed or strongly agreed that the case study strengthened each of these areas.

Furthermore, students had the opportunity to complete a free text box question related to Figure 6. In addition to their responses, a majority of respondents (95%) reported that the case study presentation increased their subject specific knowledge related to their discipline, whilst also deepening their understanding of the role of multidisciplinary teams.

### Post Survey Open Ended Questions

**FIGURE 7:**
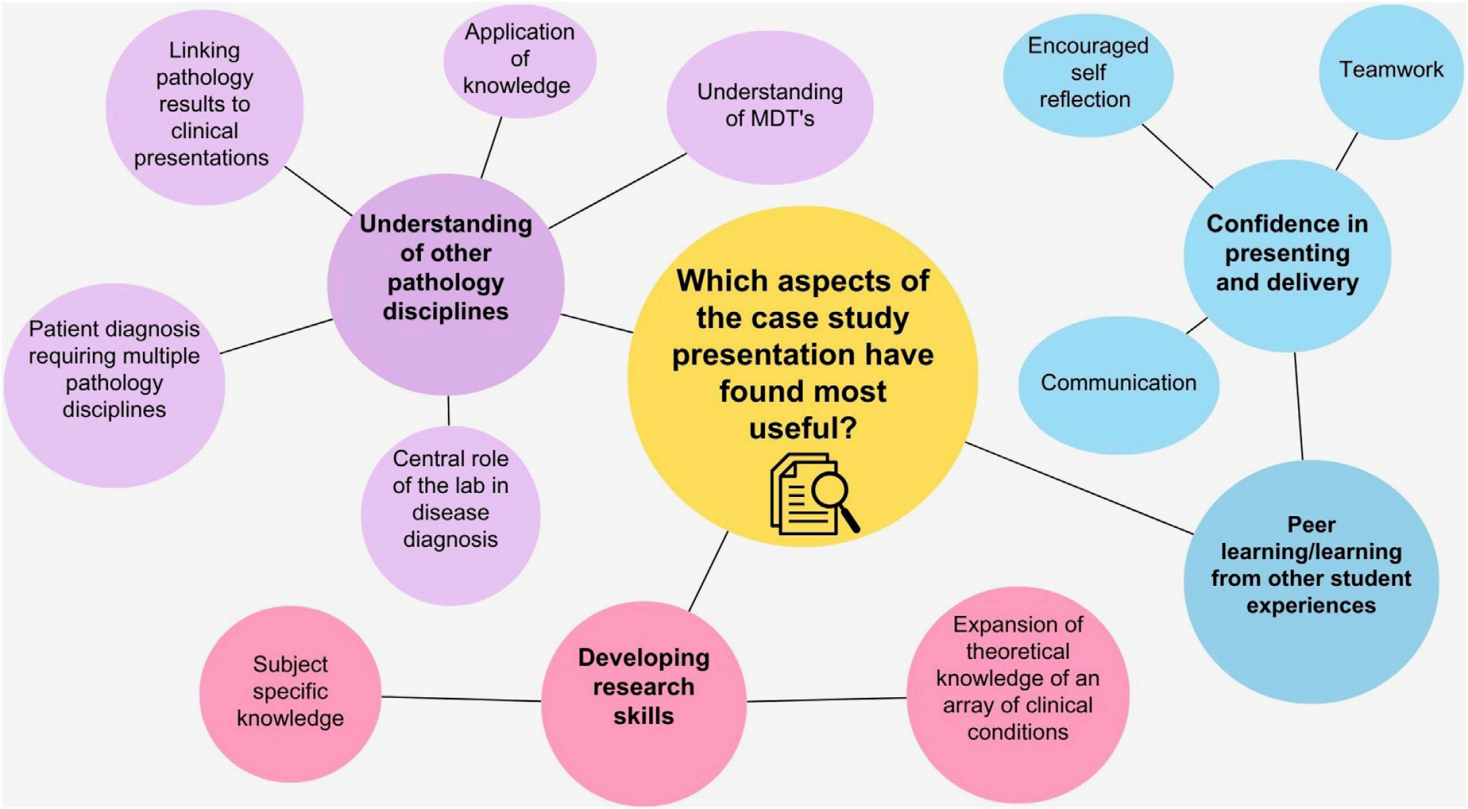
A thematic analysis of open text responses to the aspects of the case presentation that students found most useful. This question was included in the post intervention survey. Fifteen major themes were identified, and the occurrence of each theme is represented by the size of each circle in the schematic. Several students open-text response contained more than one theme.

In the post-case study workshop survey, students were asked what aspects of preparing and delivering the case study presentation they enjoyed and found to be most useful (Figure 7). Fifteen major themes were identified, with the four most prominent themes being:

• Development of research skills
• Understanding of other disciplines
• Peer learning/learning from other student experiences
• Confidence in presenting and delivery

Specific comments from students included:

“Researching has been invaluable in gaining more in-depth understanding. Presenting has helped me build my confidence”

“Gaining valuable knowledge on patient diagnosis through different laboratories and also how you have to merge and require other laboratory testing to treat a patient”

“Doing the research in a multidisciplinary team, working together to solve a case”

“I found the research aspect most enjoyable especially liaising with staff in different departments to hear about their contribution to a patient’s treatment process”

“you learn so much from case presentations and being able to watch a range of different presentations covering lots of diseases, which I used to reflect my own presentation including what went well and didn't go so well”

“Pulling a case study together across departments and reading medical reports has helped me deepen my understanding in Biomedical Science”

“I enjoyed learning about other diseases from different disciplines from the other presenters”

“I really enjoyed following a single patients journey from the onset of the health implication all the way through to the patients recovery”

“Using this opportunity to speak to Clinical scientists, Consultants etc people outside the lab that have roles in the patient pathway”

**FIGURE 8:**
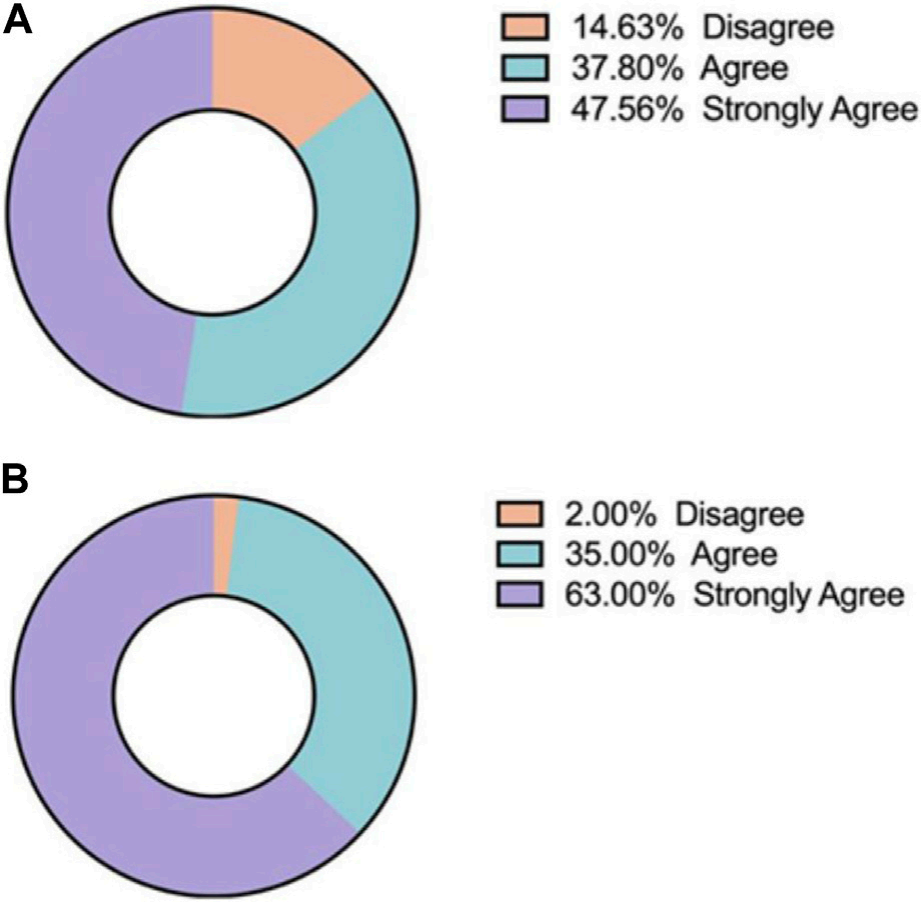
The majority of students agreed that **(A)** they would like to see more of their Biomedical Science course delivered through case presentations and **(B)** skills such as patient communication should be integrated into Biomedical Science courses.

Concluding the questionnaire, students were asked to reflect on the effectiveness of case study presentations and whether undergraduate degree programmes should introduce students to case study work, either sooner or make it an integral component of the taught material. A total of 84% of respondents either strongly agreed or agreed with this statement. Equally, students were asked to consider the importance of patient communication awareness, to which 98% of respondents either strongly agreed or agreed that patient communication should be integrated into Biomedical Sciences courses (Figure 8).

#### Feedback From Pathology Training Leads

Training Officers were asked to provide comments regarding the combined placement workshop provision, training sessions and the collaborative network within the West Midlands. Feedback was positive, and some comments included:

“The student placement workshops are of great benefit both to the placement student and the Training Officer. The student has the opportunity to meet and get to know fellow placement students from different universities and by attending the workshops they have some evidence to meet the required standards in the portfolio. This is particularly useful giving them a case study that they have to present to their peers and the workshops provide them with opportunity to gain learning with and from other healthcare professionals in order to meet the required standard in the portfolio” ∼ **Senior Training Lead Clinical Chemistry**

“The TTT sessions and combined placements workshops have reduced disparity across employers to ensure consistency in the delivery of training and assessment. Over the years the combined placement workshops have developed and enhanced considerably due to this development of a strong collaborative working with Healthcare professionals within the West Midlands” **∼ Training Officer Lead in Blood Sciences**

“Following the train the trainer sessions I have been able to introduce new elements into my training plans that have aided both students working in the laboratory and practitioners. I now ensure students visit clinics on placement, so they know the importance of the test result for the patient. I like how the combined placement workshops align to different sections of the portfolio and provide students with a range of evidence pieces” ∼**Training Lead in Microbiology**

## Discussion

The IBMS registration portfolio is a vital tool that underpins professional growth, compliance with Standards of Proficiency and ultimately HCPC registration [1]. The portfolio functions as a prerequisite to work within the NHS as a Biomedical Scientist, bolstering career opportunities whilst contributing to the integrity of healthcare services. Local NHS pathology laboratories and HEIs within the West Midlands have forged a unique symbiotic partnership to enable Training Officers to successfully support trainee Biomedical Scientists to gain registration during their placement year, consequently making them verified to work within the NHS. It is, therefore, imperative that students embarking upon a placement opportunity are well prepared but equally supported during the placement to ensure all-rounded development and successful verification. In a bid to achieve this, HEIs within the West Midlands group have designed a series of combined placement workshops, delivered throughout the placement year, aiding students to collect evidence which can be used to support the registration portfolio.

Feedback from Training Officers on case presentations from previous years highlighted that students were not including clinical data effectively. Therefore, academic leads decided to develop the existing case study structure by incorporating a pre-survey to identify students’ preconceptions of the importance of including clinical data (Figure 3). One of the major themes identified from this was students felt clinical data was core to treating patients effectively. This was followed by an interactive workshop that covered the requirements of creating and presenting a medical case presentation. Students were provided with a list of section headings that they needed to include as part of the case study construction, with the inclusion of clinical data from their laboratory being highlighted as a particularly important component. Students had the opportunity to choose a case that interested them and for which they were able to access details such as the patient history, clinical notes, diagnosis details and the treatment patients required. Although the Quality Assurance Agency for Higher Education (QAA) have guidance on subjects that need to be taught as part of a Biomedical Science degree, the disease conditions covered within these modules may vary depending on the academic expertise [9]. Through this placement workshop students were able to learn about a magnitude of clinical conditions across the pathology disciplines that they may not typically cover in an undergraduate degree (Figure 4). The data comparing student responses pre- and post-case study presentations highlights that students who engaged with the case study workshops reported an increase in self-perceived confidence in; understanding diseases and disorders diagnosed in the laboratory, linking test results to pathological conditions, delivering case studies/medical presentations, confidence in public speaking and critical thinking (Table 2). Furthermore, quantitative analysis revealed that students also deemed case presentations as an effective means of establishing links between theoretical concepts and practical application, whilst improving students’ understanding of the patient pathway (Figures 5, 6).

**TABLE 2:** A Wilcoxon signed-rank test to compare student responses pre- and post-case study presentation.

Question	Survey	*N*	Median	Interquartile range	Significance
How confident are you in your understanding of the range of diseases/disorders diagnosed in your laboratory?	Pre	48	2	1	*p* = < 0.0001
	Post	48	4	1	
How confident are you linking a test result produced in the laboratory to a disease/disorder?	Pre	48	3	1	*p* = < 0.0001
	Post	48	4	2	
How confident are you communicating/speaking in public?	Pre	48	2	1	*p* = < 0.05
	Post	48	3	2	
How confident do you feel about giving a case presentation to peers and colleagues?	Pre	48	2	1.5	*p* = < 0.001
	Post	48	3	2	
How confident do you feel in your ability to structure a medical case presentation?	Pre	48	2	2	*p* = < 0.0001
	Post	48	4	2	
How would you rate your critical thinking?	Pre	48	2	1	*p* = < 0.0001
	Post	48	2	0	

*N* = number of responses. Mean = Mean of Likert response on a scale of 1–4.

A plethora of pedagogic literature elaborates on the potential benefits of adopting case studies into higher education learning [12–16]. More specifically, case studies are one of the most frequently used teaching and learning activities within biology [17, 18]. Students enrolled on a Biomedical Science degree must be able to demonstrate proficiency in interpreting clinical case studies and be able to apply their subject knowledge regarding the aetiology, symptoms and laboratory results of a specific condition, in order to reach a suitable diagnosis and suggest appropriate treatment [9]. In addition, the HCPC highlight the importance of incorporating case studies within the Biomedical Science curriculum (SOP 10.2) [2]. The implementation of case studies functions to serve both accreditation requirements but also a vital tool in developing well-rounded undergraduate students.

### Post-Survey Thematic Analysis

In addition to identifying the transferable skills students developed through the case presentation, academics wanted to better understand the aspects of the case study presentation that students enjoyed the most (Figure 7). Through thematic analysis, one of the most prominent themes identified was that students enjoyed developing their research skills. The development of research skills remains a central component of a Biomedical Science degree [19], particularly within the final year undergraduate research projects [9]. In addition, research and competence-based skills are much sought after by graduate employers [20].

As part of developing research skills, students highlighted that the case study presentations improved their critical thinking skills and overall, the series of placement workshops enhanced student performance in completing the IBMS registration portfolio. Literature suggests that case studies allow students to develop their critical analysis skills and improve student performance [21]. Other work has highlighted that case study presentations encourage students to become reflective practitioners and enhance their problem-solving skills, in addition to allowing students to participate in group discussions [22, 23]. The case study presentation provided students with an opportunity to reinforce their subject-specific knowledge in addition to expanding their understanding of a range of clinical conditions that they may not have encountered in their degree programme. Biomedical Science undergraduate degrees require students to apply their subject knowledge to the interpretation of clinical case studies [14]. It is acknowledged that the quality of the patient’s information delivered during a case presentation is determined by the extent to which the presenter has understood the clinical problem [24].

Transferable abilities such as critical thinking, problem-solving, and communication hold significant value across the NHS. While a Biosciences degree yields students with a solid grounding in scientific know-how and specialized technical skills pertinent to the field, transferable proficiencies extend beyond domain-specific knowledge. These proficiencies empower individuals to adjust, excel, and contribute proficiently in diverse professional settings. An assessment by Noah and Aziz underscores a notable contrast between the proficiencies essential for students to stand out in the job market and those prioritised by HEIs in their curriculum [25]. The Government Office for Science has issued a report offering foresight into the trajectory of skills advancement and continual learning [26]. The report accentuates that the current work landscape is organized around profiles or roles that necessitate, alongside scientific and technical acumen gained through university education, the cultivation of a range of competencies. Correspondingly, evidence suggests that the swiftness with which graduates acclimate to their professional roles hinges on the calibre and kind of proficiencies amassed during their formal education.

Case studies promote active learning and facilitate the development of both analytical and critical thinking skills [12, 16, 27, 28]. Critical thinking is an essential skill of any undergraduate student and is a necessary skill of Biomedical Scientists, who use critical thinking to interpret laboratory test results and to problem solve [14, 29]. Furthermore, the public presentation of the case study has improve students confidence in public speaking, which is a sought out in the job market [25, 26]. Case studies also enable students to see the relevance of a given topic, and remember detail and facts [30], whilst helping to improve their knowledge and confidence [31]. Case studies are also a reflective opportunity and the use of short case study presentations as featured within this study have several advantages and have been shown to improve the clinical reasoning processes of the presenter. Advantages include [1] shortening the presentation requires abstraction of information, possibly leading to better problem representation [2]; it is time-efficient; and [3] it stimulates more informal interactions with the facilitator and the audience [24]. Case studies and scenario-based activities are key components of active learning and increase a student’s knowledge and understanding [32, 33]. Students should be supported to develop their skills with case presentation during their development as autonomous practitioners as suggested within this study.

### Multidisciplinary Teams Within the NHS

Another major theme identified was that students enjoyed acquiring an understanding of other pathology disciplines outside of their assigned placement laboratory. The role of a multidisciplinary team is to bring together healthcare professionals from different fields and expertise to diagnose and treat patients [34, 35]. In preparation for the case study workshop, students were encouraged to gain permission where possible to attend a multidisciplinary team meeting relating to the case they were presenting. Literature suggests that regular multidisciplinary team meetings help to deconstruct the complex nature of disease conditions [36]. Effective collaboration and clinical proficiency play crucial roles in clinical positions, contributing to the reduction of unjustified differences in care, enhancement of patient safety, mitigation of healthcare disparities and preservation of resources. Relatively recently, NHS pathology laboratories have aimed to meet these Standards of Proficiency by aligning their workflow into “super-labs,” essentially housing a variety of pathological disciplines into one building. This approach to delivering clinical care highlights laboratories no longer work *in silo*, therefore, to effectively diagnose a condition numerous disciplines work in unison to deliver effective healthcare. With an increasing emphasis on working collaboratively in healthcare, the importance of it is highlighted through the delivery of case study workshops.

The case study platform encouraged placement students to interact with a wide range of professionals across the hospital setting and allowed them to meet HCPC Standard 9.3 concerning *contributing effectively to work undertaken as part of a multidisciplinary team* [2]. This reminded students that a patient diagnosis is reliant upon multiple pathology disciplines working together to treat and diagnose a patient. It also highlighted to students the specialisms and opportunities available to them following graduation.

Furthermore, students enjoyed learning from their peers. Peer learning is defined as being a collaborative approach to learning and teaching involving students learning with and from each other [37]. Literature suggests that peer learning is a widely used and well-known tool often used effectively in higher education to facilitate deeper processing and long-term retention of knowledge [38]. Peer learning has also been shown to help alleviate anxiety in students who are presenting and many students who participated in the case study workshop highlighted upon reflection that they felt more confident in presenting [39].

The data presented here corroborates the critical need to infuse soft skills into student development and has been demonstrated by the inculcation of case study learning into placements. This frontward approach embodies the core principles of the NHS Long Term Workforce Plan [40] which pragmatically aims to reform NHS organisations by enhancing efficiency through varied work methods and training approaches, constructing more versatile teams with adaptable competencies, and transforming education and training to produce a larger workforce.

This case presentation workshop data demonstrates that not only did students enhance their scientific knowledge and transferable skills but they also enjoyed engaging in this activity as part of their educational journey. As academics it is difficult to always find the balance between creating necessary tasks for students to meet learning objectives and for those assessments to be engaging and interesting to students. Consequently, students reported that they would like the inculcation of case study presentations, into their respective undergraduate programmes, as it was considered an effective learning strategy, particularly in the context of clinical modules (Figure 8).

### A Novel Symbiotic Partnership the West Midlands

Several healthcare-oriented courses such as Medicine, Nursing, Midwifery, and Audiology incorporate obligatory placements as an integral part of their degree programmes. It is worth noting that while compulsory placements are a common feature in the healthcare setting, placements are not mandatory for Biomedical Science courses. Placements constitute a pivotal component of the educational journey, facilitating students in acquiring practical skills and knowledge that not only align with their academic pursuits but also foster their professional growth. The duration of these placements varies according to the specific requisites of each course. Securing positions for trainee Biomedical Scientists poses challenges primarily stemming from limitations in laboratory capacity, constraints on training time allocated for students, the availability of experienced trainers and more recently no allocated funding for BMS students on placement.

Training leads in the West Midlands, identified the pertinent training concern and a lack of graduates applying with necessary skills to work as BMS and subsequently initiated the establishment of the West Midlands Regional Training Group (WMTO). Over time, this initiative culminated in a collaborative partnership with HEIs within the local vicinity. The local hospitals identified training leads and were able to offer several placement opportunities which were advertised to local universities through the WMTO. Training officers have stated “*The HEI’s collaboration with each other and the local hospitals has enabled us to produce some excellent and extremely employable Biomedical Scientists*.”

### Limitations and Future Work

The number of placement opportunities varies annually and is dependent upon the laboratory capacity to host students. On average there are approximately 20–30 vacancies that are advertised, thus the number of students participating in the workshops is naturally impacted. Although all students completing an Applied Biomedical Science placement have to attend the compulsory combined placement workshops, completion and return of the survey was optional. Over the last 3 years, a total of 71% of respondents completed and returned the surveys, with surveys usually generating a response rate of 30%–40% [41]. Perhaps offering financial incentives would further increase the number of survey responses collected. Furthermore, students are undertaking unpaid placements and would welcome financial incentives [42].

As part of the post-case study survey, 98% of respondents wanted patient communication to be integrated into Biomedical Sciences courses. Despite the challenges of recruiting patient service users for Biomedical Science, Aston University has successfully created a service user event that includes patients, consultants and care providers in their curriculum [19]. Incorporating patients into the curriculum reinforces to Biomedical Scientists that each result is linked to the diagnosis or treatment pathway. Similarly, the inclusion of clinical case study presentations also highlights the importance of test results for the patient. Many students also presented their case studies at lunchtime seminars at their NHS Trust to a wide audience of Consultants, Medics, Biomedical Science staff, Nurses and healthcare workers, receiving outstanding feedback for their contributions. Therefore, we encourage all accredited Biomedical Science programmes to create opportunities to include patient voices and emphasise the importance of pathology test results within the patient journey.

Whilst the current combined placement programme is extensive and covers numerous HCPC SOPs (Figure 1), the West Midlands Training Officers have indicated that they would like universities to develop an interprofessional learning (IPL) workshop to foster multidisciplinary collaboration and help meet associated HCPC SOPs. Therefore, plans are underway for 2023–24 to include an IPL session discussing diabetes that incorporates Nurses, patients and Biomedical Scientists. This will be valuable in creating an opportunity for multiple professionals to work together to effectively treat patients. Furthermore, the schedule of workshops is re-examined annually to ensure they are meeting the required HCPC SOPs.

The model presented within the West Midlands successfully produces graduates who are equipped with the necessary pathology knowledge, skills and a completed IBMS registration portfolio. This partnership between HEIs and local NHS trusts within the West Midlands is a unique approach created to support students through this process. This allows graduates to register with the HCPC, thus addressing the skills shortage in the workforce. We encourage other regions to create training officer groups and to develop strong partnerships between local HEIs and pathology laboratories, with the end goal of establishing a fruitful placement programme.

## Conclusion

Combined placement workshops have formed an integral part of the Applied Biomedical Science placement journey over the last 10 years within the West Midlands. Case study presentations are a valuable teaching and learning tool to nurture and develop key transferable skills and competencies in conjunction with Biomedical Science expertise. Through engaging in the combined placement workshops and completing training in an NHS pathology laboratory, graduates in Biomedical Science elevate their attractiveness to potential employers, with a notable emphasis on the NHS. The innovative approach adopted in the West Midlands involves a collaborative model that effectively prepares graduates with essential pathology knowledge, skills, and a completed IBMS registration portfolio. This study highlights a successful framework for a collaborative partnership with local NHS trusts that has allowed the completion of numerous pathology placements and could be adopted by other HEIs delivering accredited Biomedical Science courses.

## Summary Table

### What Is Known About This Subject

• Registered Biomedical Scientists need to have completed the IBMS registration portfolio.
• Many universities offer an integrated Biomedical Science programme in which students complete the portfolio on placement.
• Across the UK there are not many examples of strong relationships between HEIs and local NHS laboratories.

### What This Paper Adds

• Showcases a unique collaborative partnership between four HEIs and local pathology laboratories in the West Midlands.
• The revised case study combined placement workshop meets HCPC SOPs and fosters development of key transferable skills.
• The combined placement workshop series develop skills and knowledge required to complete the IBMS registration portfolio.

## Concluding Statement

This work represents an advance in biomedical science because it showcases a unique partnership between HEI’s and NHS laboratories allowing successful placement completion for trainee BMS.

## Data Availability

The raw data supporting the conclusion of this article will be made available by the authors, without undue reservation.
