# Supplementary material for: Measuring the Impact of Incorporating Case Study Presentations Into Applied Biomedical Science Placement Workshops for Trainee Biomedical Scientists
Source: Br J Biomed Sci. 2024 Feb 20;81:12017. doi: 10.3389/bjbs.2024.12017 (PMC10912474; doi:10.3389/bjbs.2024.12017)
Supplement: Supplementary file 1 [file DataSheet1.DOCX]

The following questions were presented on the survey to students pre and post placement workshop:

Pre-placement workshop survey

1. Please confirm your consent to the statements above.

2. It would be helpful for us to link your pre-intervention and post-intervention responses for the research study. We don't want you to be identifiable by the data you provide but this question is just for linking responses. What is the name of your primary school?

3. Which University do you attend?

4. What are the different disciplines that make up pathology?

5. How confident are you in your understanding of the range of diseases/disorders diagnosed in your laboratory?

6. How confident are you linking a test result produced in the laboratory to a disease/disorder?

7. Have you previously presented a PowerPoint or poster presentation to an audience?

8. Do you have any experience of presenting to a virtual audience?

9. What makes PowerPoint slides visually attractive to an audience?

10. How confident are you communicating/speaking in public?

11. How confident do you feel about giving a case presentation to peers and colleagues?

12. How confident do you feel in your ability to structure a medical case presentation?

13. List what you think needs to be included in a case presentation

14. How important is the patient for a Biomedical Scientist working in the laboratory?

15. How would you rate the importance of each of the following in the patient pathway? (select from 1=most important 4=least important)

15.1. The patient

15.2. The doctor

15.3. The nurse/phlebotomist

15.4. Biomedical Scientist

16. Soft skills such as communicating with patients should be delivered during a Biomedical Scientist's undergraduate course

17. A Biomedical Scientist plays an important role in contributing to patient care

18. Without Biomedical Scientists, initiating diagnosis and treatment decisions for patients would be impossible

19. How important is clinical data interpretation when presenting a case study?

20. Critical thinking can be defined as “the objective analysis and evaluation of an issue in order to form a judgement” How would you rate your critical thinking?

21. How well do you feel that critical thinking and problem solving have been effectively covered in your undergraduate studies to date?

22. What skills do you think you need to deliver an effective presentation?

23. Why is clinical data interpretation included as part of a case presentation?

Post-placement workshop survey

1. It would be helpful for us to link your pre-intervention and post-intervention responses for the research study. We don't want you to be identifiable by the data you provide but this question is just for linking responses. What is the name of your primary school?

2. Which University do you attend?

3. How confident are you in your understanding of the range of diseases/disorders diagnosed in your laboratory?

4. How confident are you linking a test result produced in the laboratory to a disease/disorder?

5. What makes PowerPoint slides visually attractive to an audience?

6. How confident are you communicating/speaking in public?

7. How confident do you feel about giving a case presentation to peers and colleagues?

8. How confident do you feel in your ability to structure a medical case presentation?

9. List what you think needs to be included in a case presentation

10. How would you rate the importance of each of the following in the patient pathway? (select from 1=most important 4=least important)

10.1. The patient

10.2. The doctor

10.3. The nurse/phlebotomist

10.4. Biomedical Scientist

11. A Biomedical Scientist plays an important role in contributing to patient care

12. How important is clinical data interpretation when presenting a case study?

13. Critical thinking can be defined as "the objective analysis and evaluation of an issue in order to form a judgement". How would you rate your critical thinking?

14. What skills do you think you need to deliver an effective presentation?

15. Presenting the case study has improved each of the following:

15.1. (A) Your ability to communicate your knowledge of scientific concepts orally?

15.2. (B) Your understanding of the underlying biological basis of the disease in the case?

15.3. (C) Establish the link between theoretical content and practice?

16. To what extent do you agree with the following statements:

16.1. Whilst presenting the case I deepened my understanding of the topic using online resources/books/review papers?

16.2. The case presentation exercise facilitated the development of reflective thinking and critical thinking

16.3. Following the case presentation I now have a better understanding of the whole patient pathway from sample collection, processing through to the treatment of the patient?

16.a. Please provide details of why you have provided those responses

17. The case presentation has changed my understanding of the Biomedical Scientists role in delivering effective patient care

18. I would like to see more of my Biomedical science course delivered through case- study presentations?

19. I have enjoyed engaging in this case-study task

20- please state which aspects you have found most useful/enjoyable
